# Topical gel based nanoparticles for the controlled release of oleanolic acid: design and in vivo characterization of a cubic liquid crystalline anti-inflammatory drug

**DOI:** 10.1186/s12906-021-03399-8

**Published:** 2021-09-04

**Authors:** Zhiqi Shi, Shugang Pan, Luolin Wang, Sha Li

**Affiliations:** 1 Changzhou Institute of Industry and Technology, No.28#, Mingxin Road, Changzhou, 213164 Jiangsu Province China; 2 Changzhou Institute of Technology, Changzhou, 213022 Jiangsu Province China; 3 Key Laboratory for Soft Chemistry and Functional Materials of Ministry Education, Nanjing University of Science and Technology, Nanjing, 211816 Jiangsu Province China; 4 Guangdong Provincial Institute of Traditional Chinese Medicine, Guangzhou, 510095 Guangdong Province China; 5 Wuxi Hospital of Traditional Chinese Medicine, No.8#, Zhongnan Road, Wuxi, 214071 Jiangsu Province China

**Keywords:** LCNP-based gel, Oleanolic acid, Anti-inflammatory, Permeation studies

## Abstract

**Background:**

Oleanolic acid (OA) has multiple pharmaceutical applications including anti-inflammatory activity, but low permeability of the molecule limits its widespread use.

**Methods:**

A cubic liquid crystalline nanoparticle (LCNP)-based gel was prepared as a potential topical delivery system for OA. The LCNP-based gel was optimized using rheological, drug release kinetic, and ex vivo permeation studies.

**Results:**

The studies showed that the OA was trapped in the interior of the LCNP with a crystal form of Pn3m space. The optimized LCNP formulation performed well using in vitro release studies for up to 12 h (85.49 ± 0.21%). Ex vivo permeation studies showed that the LCNP-based gel formulation was superior to a standard gel formulation. The r^2^ value from the Peppas equation indicated good linearity, but showed irregular (non-Fickian) diffusion, suggesting that drug release was controlled by multiple processes.

**Conclusions:**

In this study, OA-loaded LCNPs were prepared by the precursor method, resulting in a well-characterized OA-LCNP gel preparation. The gel was shown to be effective in a rodent carrageenan-induced hind paw inflammation model with sustained efficacy after a single application.

## Introduction

Many plants in nature have been proven to have a variety of pharmacological activities, prompting scientists to find ingredients with specific activities in them for better treatment of various diseases. Many triterpenoids including OA have been shown to have a wide range of pharmacological activities [1] including being an antioxidant, hypoglycemic, anti-inflammatory, antibacterial, and hepatoprotective [2–8]. The findings of the referenced study revealed OA to possess dose related anti-inflammatory activity in a variety of test models including carrageenan-induced oedema in rats [5]. It has been reported that OA could inhibits mouse paw edema and effectively increase the number of Treg cells and the expression of IL-10 and IL-35 [9, 10]. In addition, OA also caused a pleiotropic antibacterial effect as they were able to influence various cellular functions including induction of the stress response [11]. It may be concluded that OA, whose spectrum of anti-inflammatory activity appears to be different from classical NSAIDs, would be of therapeutic value [12, 13].

However, OA is a class IV drug according to the Biopharmaceutics Classification System, and has an absolute oral bioavailability of only 0.7% because of its low permeability (Papp = 1.1–1.3 × 10^− 6^ cm/s in the apical to basolateral direction at 10 and 20 μM) and low aqueous solubility (< 1 μg/mL) [14, 15]. Several approaches including recrystallization [16], solid dispersion [17], nano-emulsions [18], and drug–phospholipid complex techniques [19] have been used to attempt to improve the bioavailability of OA.

Cubic liquid crystal nanoparticles (LCNP) are now being investigated as a universal carrier for drug delivery [20–24] to improve the oral bioavailability of the drug because of high encapsulation efficiency, drug loading and bio-affinity [25]. In our previous study, we prepared curcumin and piperine-loaded LCNP using a precursor injection method. The results showed that the oral bioavailability of LCNP-curcumin was significantly improved [26].

Based on our previous work and referenced evidence, in this study we explored the possibility of LCNP as a carrier system for a topical formulation of OA. In addition, in this study we also evaluated the in vitro permeability of the drug through rat skin and determined the anti-inflammatory activity of the LCNP gel.

## Materials and methods

### Material

Phytantriol (purity > 98%) was purchased from TCI (Tokyo, Japan). OA was acquired from Acetar Bio-tech Inc. (Shanxi, China). Lutrol F127, propylene glycol, glycerin, carbopol 934 (CP), HPMC K4M, ethanol, were all acquired from Shanhe Pharmaceutical Accessories Co., Ltd. (Anhui, China). Sodium dodecyl sulfate was purchased from Hunan Er-kang Pharmaceutical Co., Ltd. (Hunan, China). Azone was purchased from Tianmen scientific Pharmaceutical Co., Ltd. (Hubei, China). Triethanolamine was given as a gift from Jiangxi α-Hitech scientific Pharmaceutical Co., Ltd. (Jiangxi, China). Ultrapure water was produced by using a Milli-Q purification system from Millipore (Massachusetts, USA). Acetonitrile and methanol (both HPLC grade) were purchased from Merck (New Jersey, Germany). Standards of OA (CAS: 508–02-1, > 95%) were purchased from Nation Institutes for Food and Drug Control (Beijing, China). All materials were used as received.

### Animals

Male rats (weighing 180–220 g) were obtained from Guangdong Medical Laboratory Animal Center, and were housed under 12 h: 12 h light-dark cycle, constant temperature of 22 ± 2 °C and 40–50% humidity. All laboratory animals had free access to water and food. The study was carried out in compliance with the ARRIVE guidelines. The experimental protocols and treatments were all approved by Ethics Committee of Guangdong Provincial Institute of Traditional Chinese Medicine.

### Methods

#### Screening of excipients

**Fig. 1:**
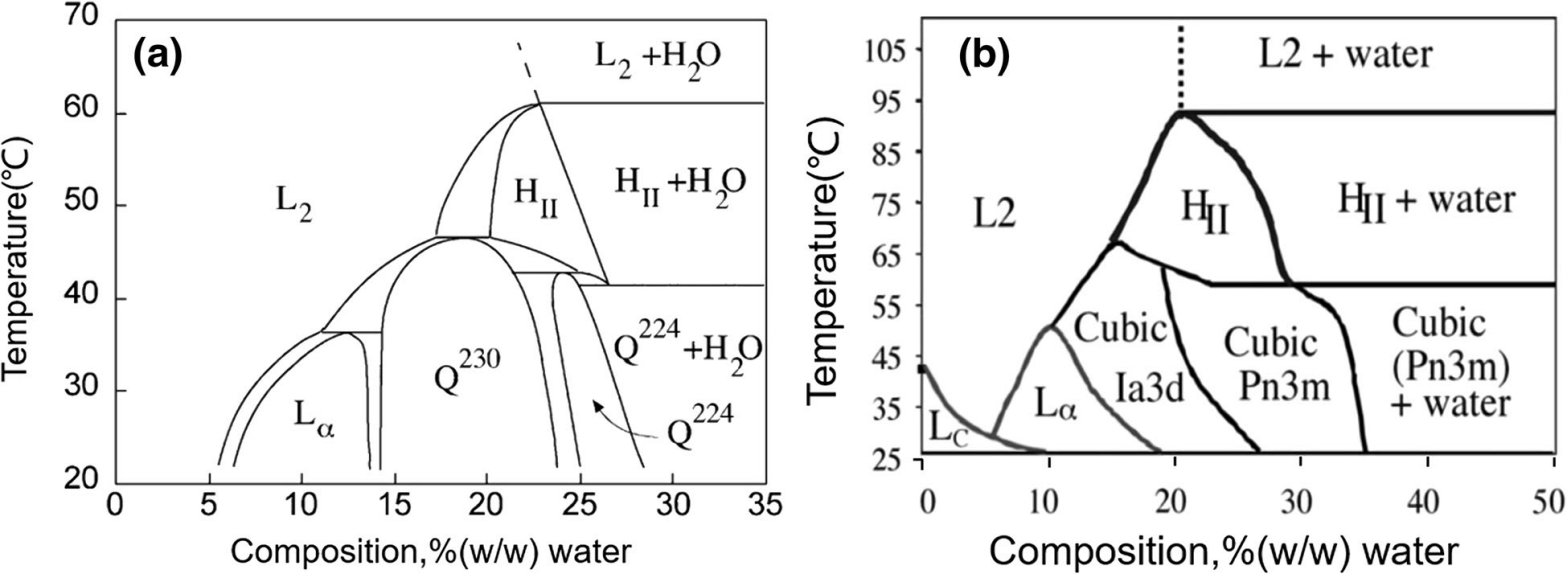
Binary phase diagram of phytantriol-water (**a**) and glycerol monooleate-water (**b**)

When developing the LCNP system, suitable liquid, lipid and surfactants were selected for use, and were generally considered safe (GRAS). At present, the excipients used to prepare LCNP mainly include phytantriol and glycerol monooleate(GMO). These two materials have different ternary phase diagram regions, and the conditions for forming cubic liquid crystals are also different (Fig. 1) [32, 33].

OA and excipient (Phytantriol or GMO) were weighed and dissolved in 2.5 mL of absolute ethanol simultaneously with sonication at 80% of maximum power for 30 min. The organic phase was added dropwise to the aqueous phase 20 mL containing stabilizer F127 under agitation at 500 rpm for 3 h to form a crude dispersion, which was then sonicated (Misonix XL2000, Misonix Inc., Farmingdale, N.Y.) in a pulsed mode (0.5 s pulse interrupted by 0.5 s interrupt) at 40% of maximum power for 10 min to produce a milky dispersion and to reduce particle size.

#### Surfactant screening

In order to prepare stable LCNPs, two surfactants (Lutrol F127 and Vitamin E Acetate) were screened.

#### Preparation of OA loaded LCNP

The nanoparticles were prepared using the precursor method described in our previous study [26, 27]. Drugs and excipients were weighed according to the prescriptions in Table 1. The phytantriol and OA were dissolved in 2.5 mL of absolute ethanol simultaneously with sonication at 80% of maximum power for 30 min. The organic phase was added dropwise to the aqueous phase (20 mL) containing stabilizer F127 under agitation to form a crude dispersion, which was then sonicated in a pulsed mode (0.5 s pulse interrupted by 0.5 s interrupt) at 40% of maximum power for 10 min to produce a milky dispersion and to reduce particle size. In addition, LCNP containing OA with loading amounts of 6, 8 and 10%, respectively, were prepared and their effects on encapsulation efficiency were investigated. The effects of different stirring speeds (500, 1000, 1500 rpm) and homogenization cycle (3, 6, 9 cycles) were optimized based on the measurements of particle size and encapsulation efficiency, respectively. The OA-loaded LCNP dispersion obtained was freeze-dried to be a dry and free-flowing powder.

**Table 1:** Different Formulations for OA-loaded LCNP

S.No.	Formulations parameters	Formulations		
		OAF1	OAF2	OAF3
1	Phytantriol (mg)	70	80	90
2	F127(mg)	30	20	10
3	OA (mg)	6	8	10

### Particle size, micrographs and crystal cell parameter measurements of OA-loaded LCNP

The size of LCNP was measured using dynamic light scattering (Nano patica SZ100, H field, Iwate Prefecture, Japan); we used an average of five measurements. Micrograghs of the nanoparticles were investigated by scanning electron microscopy (SEM; 1430VP, Leo, Sauerlach, Germany) [21]. In determining the size of the nanoparticles, the cubic phase nanoparticle suspension was first placed on silicon water through a capillary tube, then allowed to dry at room temperature. Thereafter, they were coated with platinum for 2 min in the ion sputtering instrument. The coated samples were measured at an acceleration voltage of 15 kV. Average particle size and polydispersity index were measured by photon correlation spectroscopy at a 90° angle at 25 °C.

Polarizing light microscopy (BX41P, Olympus, Tokyo, Japan) with a (MATS-U55S, Tokaihit, Shizuoka, Japan) heater set between a range of 20–60 °C [21] was used in this study The crystal cell parameter measurement used a Bruker Nanostar small-angle X-ray scattering (SAXS) camera with pinhole collimation for point focus geometry. The sample was placed in the quartz capillary tube, and the optics and sample chamber were placed under vacuum to minimize air scatter. The results were normalized for sample transmission after the background was subtracted and integrated using Bruker AXS software. The diffraction patterns obtained were converted to plots of intensity versus q value.

### Zeta potential analysis of OA-loaded LCNP

The nanoparticle surface charge was measured by determining the zeta potential using a Zetasizer (3000HS Malvern Instruments, UK). The LCNP suspension was diluted with double distilled water (1:100, w/w) to obtain a uniform dispersion before testing, and the conductivity was then measured at 25 °C [28]. All values calculated were the average of three independent measurements (mean ± standard deviation).

### Drug entrapment efficiency (DEE) and drug loading (DL) of OA-loaded LCNP

Nanoparticles were embedded in a Sephadex G-50-filled microcolumn under a centrifugal force of 500 rpm for 5 min and then eluted with the same volume of ultrapure water. The eluate was obtained by centrifugation at 2000 rpm for 5 min. After repeating the same procedure for five times, the eluate was then collected and dissolved in methanol. The drug content of the eluate was measured in triplicate by a validated HPLC method. DEE of LCNP was calculated as follows: DEE (%, w/w) = Total amount of drug- Amount of drug in supernatant× 100/Total amount of drug. DL (%, w/w) = Initial drug – free drug/Mixed lipid × 100.

### In vitro release from OA-loaded LCNP

The in vitro release was measured by a dialysis method. Briefly, the nanoparticles and the suspensions were placed in the dialysis bag (Biosharp, Hefei, China) with a molecular cut off of 14 kDa, and the dialysis bag was suspended in 20 mL of release medium (containing 0.5% sodium lauryl sulfate) at 37 °C while the speed of the magnetic agitation was set at 300 rpm. 100 μL of sample was withdrawn at predetermined time interval and replaced with fresh medium. The sample content was then determined by the in vitro HPLC method. The HPLC apparatus used for the quantitation consisted of an Agilent 1200 HPLC system (Agilent, USA) with DAD detector. Compounds were separated on a Kromasil RP-C18 column (4.6 mm × 250 mm, Akzo Nobel, Stockholm, Sweden) using a flow rate of 1.0 mL/min with a fixed mobile phase [acetonitrile and 0.1 moL·L^− 1^ ammonium acetate (55:45)]. The wavelength was set at 210 nm. The injection volume was 10 μL and the retention time of OA was determined to be 27.665 min. The LCNP matrix contained the same amount of ingredients but without OA, and this was used as a blank for comparison. Calibration curves plotted for OA showed good linearity, with a correlation coefficient (r^2^) of 0.9993 in the concentration ranges tested (10.42 ~ 104.2 μg·mL^− 1^).

This analytical method has been validated in accordance with the guidelines of the International Conference on Harmonization of Technical Requirements for Registration of Pharmaceuticals for Human Use (ICH). Parameters such as accuracy, reproducibility, and stability were verified separately. The relative standard deviations (RSD) of intra and inter day analyses were < 3%, and the accuracy of the method was verified with the average recovery rates of 99.02%.

### Preparation of OA-LCNP-based gel

After optimizing the OA-LCNP method, the topical gel loaded with OA-LCNP was prepared by a dispersion swelling technique using Carbopol 934 and HPMC K4M. All excipients were weighed as shown in Table 2, and then Carbopol 934 and HPMC K4M were simultaneously dispersed in water and allowed to stand for 4 h to properly swell the polymer. OA-LCNP powder (equivalent to 0.5 mg/g) was then added to the polymer gel under constant one-way agitation to avoid air bubbles. Triethanolamine was then added to the gel mixture for crosslinking between Carbopol 934 polymer and HPMC K4M to form a gel. Then, propylene glycol and glycerin were added to the gel to balance its viscosity, azone was added to increase the permeability of the gel, and finally the pH was adjusted to a skin pH of 7.4 ± 0.1 by addition of 0.1 N NaOH [29]. In addition, a conventional OA-loaded gel (NG) was prepared in the same manner without OA-loaded LCNP.

**Table 2:** Formulation table for OA-LCNP-based Gel

S.No.	Ingredients	OA-LCNPG1(w/w)	OA-LCNPG2(w/w)	OA-LCNPG3(w/w)	NG(w/w)
1	CP 934	1.5%	1.0%	0.5%	1.0%
2	HPMC K4M	0.5%	1.0%	1.5%	1.0%
3	glycerol	10%	10%	10%	10%
4	propylene glycol	5%	5%	5%	5%
5	Triethanaolamine	2%	2%	2%	2%
7	Azone	1%	1%	1%	1%

### Physical appearance and formulation pH

Basic physicochemical parameters were screened for different formulations. The color, transparency, homogeneity and appearance of the prepared gel were visually inspected. The pH of the 1% aqueous solution of the gel was measured by a pH meter (Mettler Toledo, Switzerland). One gram per meter of gel was dissolved in 100 mL of distilled water and stored for 2 h [23]. The pH of each formulation was measured in triplicate and the average was calculated.

### Viscosity and Spreadability analysis of OA-LCNP-loaded topical gel

In the rheological properties test, all developed formulations were placed in a beaker, placed in the rotor, and then rotated at 10 rpm (25–27 °C) in a Brookfield viscometer (V-550, Thermo). To measure the spreadability of the formulated OA-LCNP gel, we introduced 1 g of gel per gram between two slides, held the pre-weighed plate over the gel and gradually added more weight until the gel stopped spreading. The final cumulative weight and total time required for gel diffusion were measured and recorded separately. The spreadability of the gel was then calculated according to the formula, i.e., the total weight applied and the gel mass were compared by the time [29]. Spreadibility = Mass× Length/Time.

### Ex vivo drug permeation studies of OA-LCNP-loaded topical gel

The measurements of drug permeation were performed using a Franz diffusion cell to evaluate the OA release profile from each formulation. The surface area of the release membrane was 1.971 cm^2^, and the receiving chamber volume was 18.54 mL. Before performing the permeability study, the abdominal skin of the rat was obtained and processed. The subcutaneous tissue was carefully removed by surgery, and the dermal side was wiped with isopropyl alcohol to remove the attached fat layer. The skin was cleaned again with distilled water and then stored at − 18 °C. During the permeability test, the skin was placed between the donor and recipient compartments of the Franz cells with the stratum corneum side facing the donor compartment and the dermis side facing the receptor compartment [30].

One gram (equivalent to 0.5 mg OA) of gel (NG, OA-LCNP G1, OA-LCNP G2, OA-LCNP G3, and NG) was added in to the donor compartment. The receptor compartment was filled with PBS (pH 7.4) (containing 0.5% sodium lauryl sulfate) and kept in full contact with the dermis layer, the temperature was controlled at 37 °C, and the speed of magnetic stirring was performed at 400 rpm. 2 mL of the receptor solution was withdrawn at an interval of 0.5, 1, 2, 3, 6, 9, 12 h. After each sampling, an equal volume of PBS was simultaneously added to the receptor compartment to maintain volume. Each sample was filtered through a 0.45 μm polyamide membrane filter (Satorius, Germany), and then the OA content was determined by HPLC. The concentration in the sample taken and the percentage of drug release of the preparations were calculated.

### Analysis of permeability OA-LCNP-loaded topical gel

For each formulation, the percentage of drug permeated through the skin (μg·cm^− 2^) was plotted as a function of time. By dividing the slope of the linear portion of the graph by the diffusion cell area (μg·cm^− 2^·h^− 1^), the drug flux (permeability) at steady state (Jss) was calculated. The permeability coefficient (Kp) was calculated by dividing Jss by the initial concentration of the drug in the donor cell (cm·h^− 1^). The enhancement rate (Er) was calculated by dividing the Jss of each formulation by the Jss of the control formulation [30].

### Drug release kinetics studies OA-LCNP-loaded topical gel

The data obtained from in vitro drug release studies were plotted as percent drug release versus time (Zero-order equation), logarithm of drug residue versus time (First-order equation), percentage of drug release versus square root of time (Higuchi model equation), and the logarithmic relationship between logarithmic drug release and logarithmic time (Korsmeyer equation) to assess drug release mechanisms. The standard values for the release mechanism are listed in Table 5.

### In vivo anti-inflammatory studies of OA-LCNP-loaded topical gel

Anti-inflammatory studies were performed in adult male Wistar rats weighing 150–250 g according to a previously reported technique [31]. Briefly, rats were randomly divided into three groups, One experimental group and two control groups (*n* = 6 per group). Group I received topical saline application (control group), Group II received commercially available mometasone furoate gel (standard group), and Group III received LCNP-based gel formulation (test group).

The left hind paw of the rat was first marked directly above the tibia-tar junction. The paw was immersed in the electrolyte column every time until the marker was fixed in order to measure the volume of the constant paw. The test formulation was applied to the left hind paw of the rat for 30 min before carrageenan-induced inflammation. The initial paw volume (Vo) of the rats was determined just prior to injection of carrageenan, and the volume increase due to fluid excretion was noted from the digital display, and then 0.1 mL of 1% carrageenan solution was injected into the saline which located at the subplantar area of the left hind paw of the rat. The paw volume (Vt) was then assessed after 1, 2, 4, 8, 12 and 24 h, respectively. The edema rate and inhibition rate of each group are calculated as follows:

$$ \mathrm{Edema}\ \mathrm{Rate}\ \left(\mathrm{E}\%\right)=\left(\mathrm{Vt}-\mathrm{Vo}\right)/\mathrm{Vo}\times 100 $$

$$ \mathrm{Inhibition}\ \mathrm{Rate}\ \left(\mathrm{I}\%\right)=\left(\mathrm{Ec}-\mathrm{Et}\right)/\mathrm{Ec}\times 100 $$

Where Ec is the edema rate of control group, and Et is the edema rate of the treated group.

## Results and discussion

### Screening of excipients

It can be seen from Fig. 1 that GMO could form LCNP with different crystal lattices below 60 °C with water content above 10%, and LCNP can also be formed by phytantriol when the water composition was above 15% with the temperature below 45 °C. As it was shown in Table 3, we found that higher entrapment efficiency was obtained with Phytantriol (68.12 ± 2.36% w/w) but with larger size particle (179 ± 13.63 nm). However, GMO resulted in smaller size particle (154 ± 11.36 nm) but with lower entrapment efficiency (59.41 ± 2.32% w/w) and drug loading(10.12 ± 0.43% w/w). Thus, phytantriol was selected as the suitable excipient.

**Table 3:** Particle size, Zeta potential, and polydispersity index of OA-LCNP

No.	Formulations(mg)			Particle size (nm) ± SD	DEE (% w/w) ± SD	DL (% w/w) ± SD
	Phytantriol	GMO	F127			
1	90	/	10	179 ± 13.63	68.12 ± 2.36	13.04 ± 0.56
2	/	90	10	154 ± 11.36	59.41 ± 2.32	10.12 ± 0.43

### Surfactant screening

From the results in Table 4, smaller size particle can be formed by Vitamin E Acetate but with lower entrapment efficiency (59.41 ± 2.32% w/w) and drug loading(10.12 ± 0.43% w/w), which might be due to the numbers of hydroxyl and the net structure of the stabilisers. F127 was chosen as a favorable stabilizer in the after studies.

**Table 4:** Particle size, Zeta potential, and polydispersity index of OA-LCNP

No.	Formulations(mg)			Particle size (nm) ± SD	DEE (% w/w) ± SD	DL (% w/w) ± SD
	Phytantriol	F127	Vitamin E Acetate			
1	90	/	10	104 ± 14.32	4.46 ± 1.24	1.02 ± 0.21
2	90	10	/	179 ± 13.63	68.12 ± 2.36	13.04 ± 0.56

### Screening of formula technology

We compared 2 methods to prepare LCNP: the precursor injection method and the solvent emulsion evaporation technique. The precursor-injection method exhibited the smallest particle size (273 ± 18.92 nm) and highest entrapment efficiency (70.12 ± 2.12% w/w) compared to the solvent emulsification evaporation technique, which resulted in a particle size of 543 ± 32.93 nm and entrapment efficiency of 45.18 ± 2.08% w/w.

### Optimization of process parameters

**Fig. 2:**
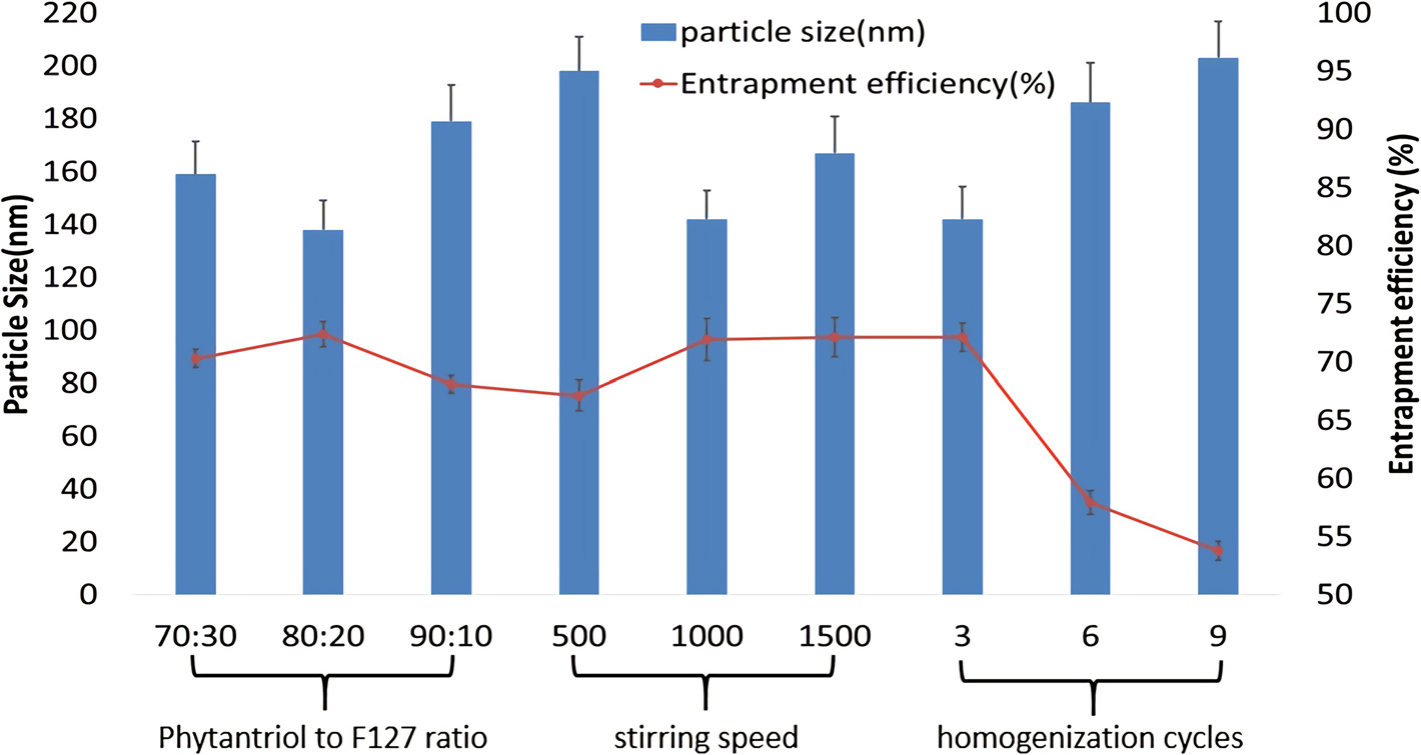
Process parameters on the basis of particle size and entrapment efficiency

Several process parameters were determined prior to preparation of the LCNP. The ratio of phytantriol to F127, surfactant concentration, agitation time, agitation speed and homogenization cycle were optimized over a wide range to enable selection of the optimum formulation. Since the quality and efficacy of cubosomes were significantly affected by the properties and concentrations of the surfactant, the ratio of phytantriol to F127 was optimized with the measurement of particle size and encapsulation efficiency [34]. The ratios of phytantriol to F127 were assumed to be 70:30, 80:20 and 90:10, respectively. The results showed that the ratio of phytantriol to F127 was 80:20 with the smallest particle size (138 ± 10.21 nm) and the optimal encapsulation efficiency of the nanoparticles is 72.39 ± 2.36% w/w, as shown in Fig. 2.

The effects of different stirring speeds between 500, 1000, 1500 rpm were screened, and 1000 rpm was chosen because it had the lowest particle size 142 ± 11.83 nm and the best encapsulation efficiency 71.93 ± 2.93% w/w as shown in Fig. 1.

In addition, stirring time between 15, 30, 45, and 60 min was also screened and it was determined that 30 min was optimal to obtain the lowest particle size. The homogenization cycle was screened based on the measurements of particle size and encapsulation efficiency for 3, 6 and 9 cycles. The results showed that as the number of cycles increased, the particle size first decreased and then increased slightly. This may be due to the fact that as the homogenization cycle increased, the system temperature gradually decreased, resulting in an increase in kinetic energy, thus the particles reassemble during preparation [23]. Six cycles achieved a minimum particle size of 142 ± 9.98 nm and an optimal drug retention of 72.17 ± 2.83% w/w as shown in Fig. 1.

When assessing evaluated parameters, we developed different formulations to take into account optimization of parameters, i.e. 0, 1 (optimization parameters), and the + 1 level was certain as given in Table 1. The LCNP loaded with OA was successfully prepared by the precursor injection method, and the parameters such as particle size, encapsulation efficiency, zeta potential and drug release in vitro were characterized. The LCNP loaded with OA showed particle size in the range of 129 ± 12.11 nm to 272 ± 21.83 nm. The minimum particle size was 129 ± 12.11 nm of the formulation OAF1, which is the certain condition for local delivery of LCNP as shown in Table 5.

**Table 5:** Particle size, Zeta potential, and polydispersity index of OA-LCNP

Formulations	Drug loaded(%, w/w)	Particle size (nm) ± SD	Zeta potential	Polydispersity index	DEE(% w/w) ± SD	DL(% w/w) ± SD
OAF1	6	129 ± 12.11	−18.3 mV	0.322	73.18 ± 3.21	12.31 ± 0.41
OAF2	8	159 ± 17.87	−21.2 mV	0.218	70.93 ± 3.28	14.12 ± 0.32
OAF3	10	272 ± 21.83	−19.9 mV	0.436	68.31 ± 2.86	13.11 ± 0.74

The particle size of OAF1 and OAF2 decreased as the concentration of the surfactant increased, but we noted an eventual increase. This may be due to an increase in the homogenization cycle and OA content. As the homogenization cycle increased, the kinetic energy of the system increased, causing particle aggregation [29]. The smaller size may help improve penetration of the drug through the biofilm and aid in targeting. These are considered to be important criteria for topical drug delivery systems that use loaded nanoparticles. In general, the particle size of the nanoparticles should be as small as possible to penetrate the skin more easily, especially in the case of LCNP. Under some circumstances, entrapment efficiency can be considered to be less important than particle size for LCNP formulation development. The values of zeta potential were in the range of − 18.3 to − 21.2 mV for all three formulations. A high zeta potential is related to the storage stability of the nanoparticles, which may indicate that the nanodispersions may not aggregate. We found that the zeta potential increased and then decreased (OAF1 < OAF2).

**Fig. 3:**
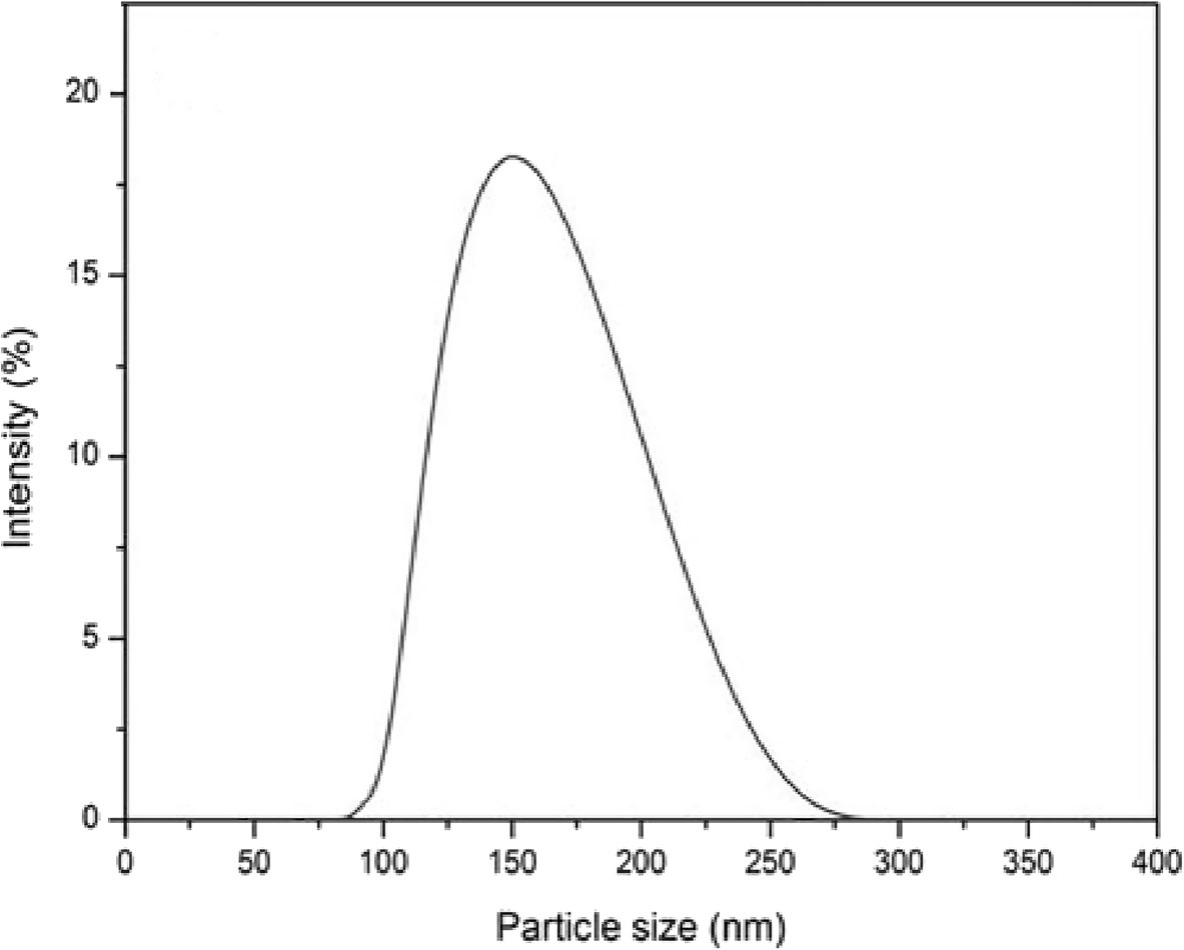
Particle size distribution of OA-loaded LCNP

**Fig. 4:**
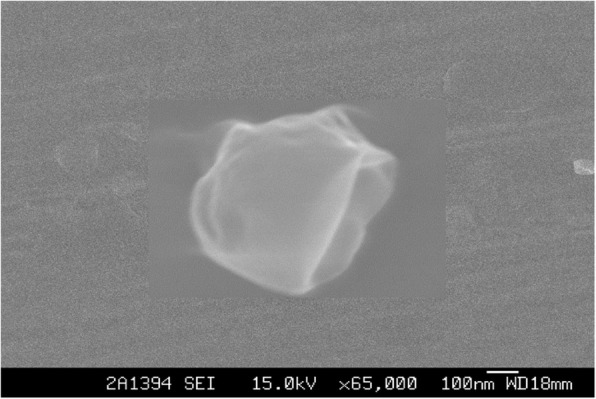
Morphology of OA-loaded LCNP by scanning electron microscope (Magnification 65,000)

Determination of the particle size and the distribution of nanoparticles require measurement of the polydispersity index (PDI). A sample having a polydispersity value of less than 0.7 indicates that the nanoparticles are substantially uniformly dispersed. The results showed that the values of the PDI of the three formulations ranged from 0.218 to 0.436, with the formula CPF2 showing a minimum PDI of 0.218. The results showed that the PDI value decreased with an increase of phytantriol and then increased. The shape and surface morphology of the LCNP were observed by scanning electron microscope (SEM). The average particle size of the cubic nanoparticles was 152 nm as determined by dynamic light scattering (DLS) (Fig. 3). The SEM image revealed that the particles were cube-shaped with Pn3m space group and stacked in 3D (Fig. 4).

**Fig. 5:**
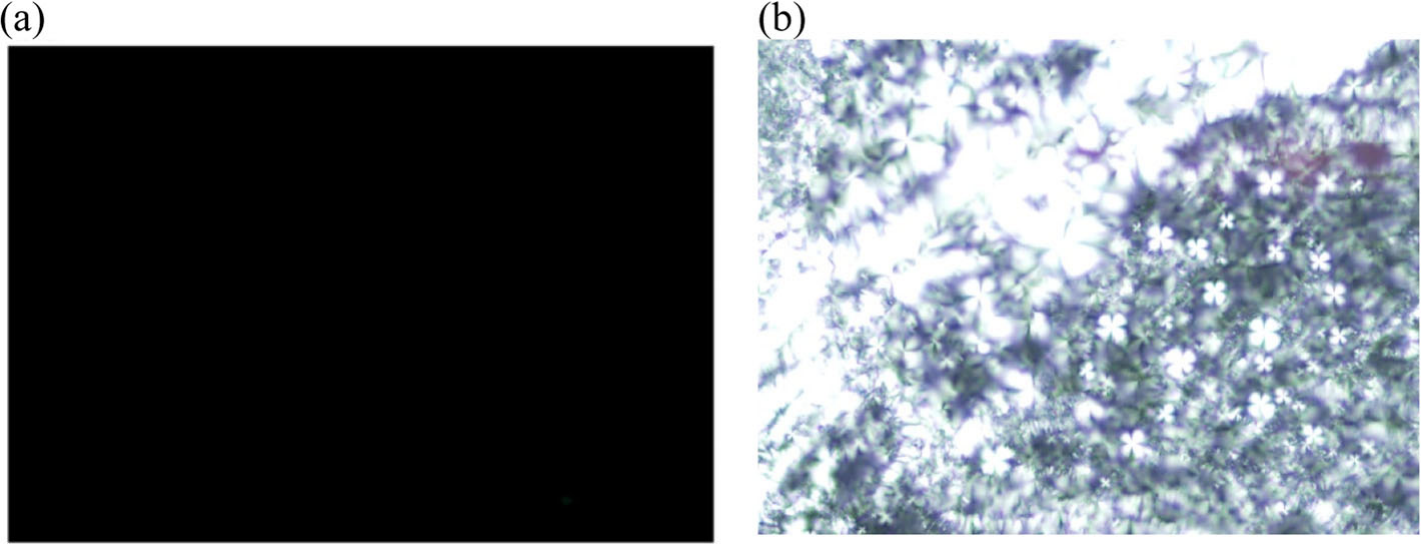
**a** Polarizing light micrograph of OA-loaded LCNP at 20 °C and **b** the cubosome at 55 °C

Due to the optically isotropic properties of LCNP, the cubic liquid crystal appeared dark at 20 °C in the field of view, which usually indicated the formation of a cubic phase. However, when the sample was heated at 55 °C, the anisotropic image appeared as a fan-like texture, indicating that the Hk phase was formed instead of the cubic phase, which was due to the transformation of the crystal structure (Fig. 5) [35] and also consistent with our previous research [26]. In addition, when the temperature drops to 20 °C, the dark field of the image reappeared which indicated the formation of a cubic phase.

**Fig. 6:**
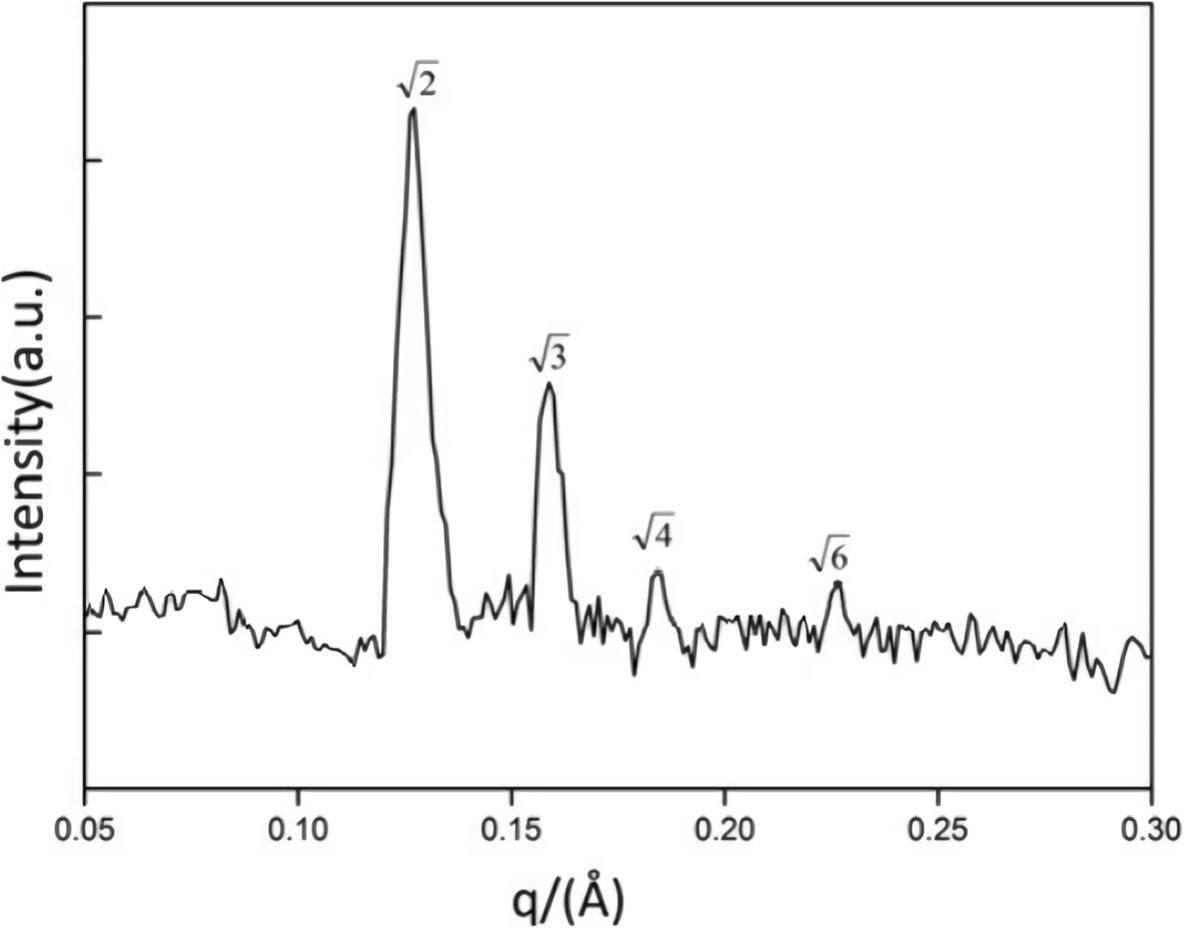
Intensity versus q plot from SAXS measurement of OA-loaded LCNP

SAXS was used to determine the internal structure of the cubosome nanoparticles. Figure 6 shows a plot of intensity versus the scattering vector and q, obtained from formulations prepared using phytantriol and F127. The OA-loaded LCNP displayed four peaks, where the first two were more intense than the second pair. The relative position of the peaks was in accordance with the bicontinuous cubic phase structure with Pn3m space group, indicative of the cubosome nanoparticles with a D-type cubic nanostructure with lattice parameters of 67.4 Å.

**Fig. 7:**
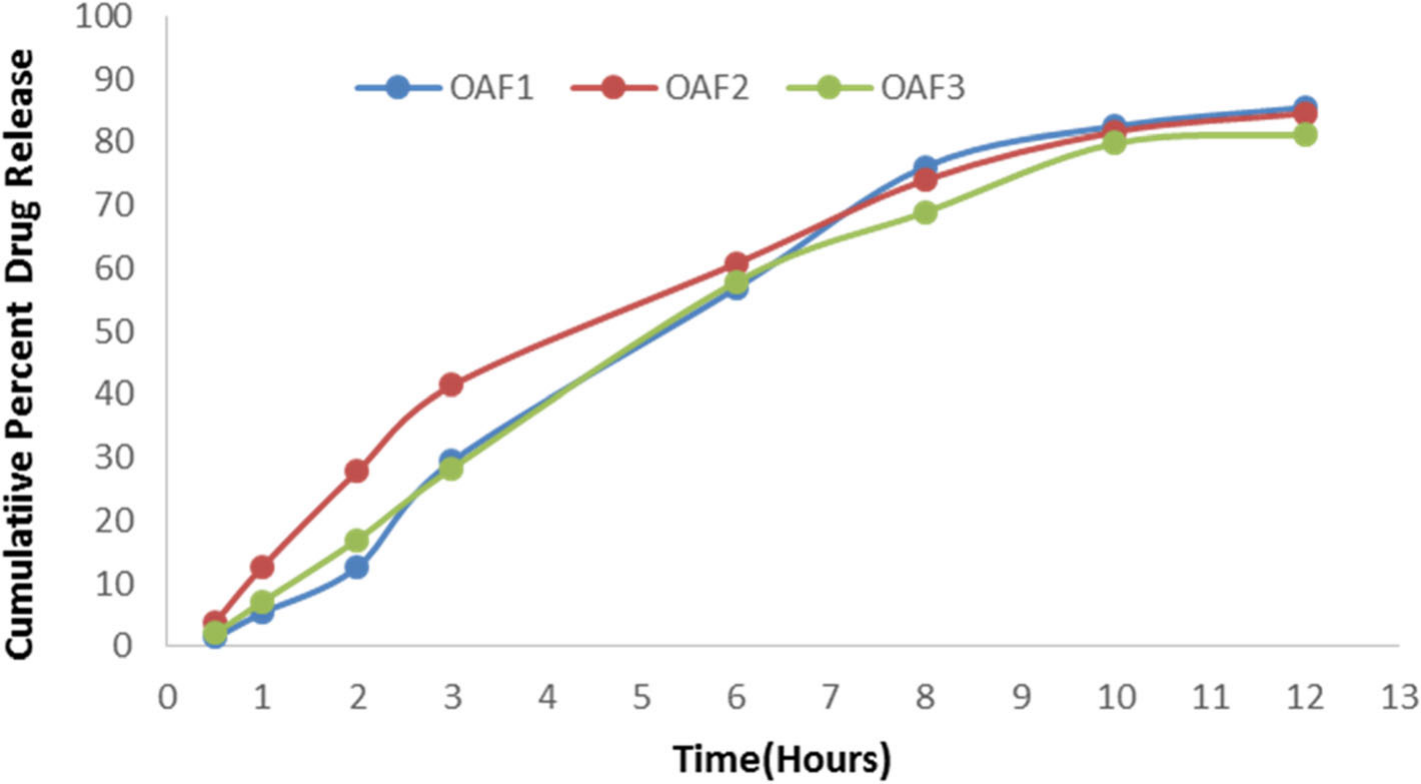
- In vitro drug release profiles of OA-loaded LCNP up to 12 h

The encapsulation efficiency of the LCNP loaded with OA was in the range of 68.31% ± 2.86 to 73.18% ± 3.21 w/w. Encapsulation efficiency is related to the crystallinity of the lipid nanoparticles; the more phytantriol in the formulation mixture, the higher with the entrapment efficiency. The drug loading of OA-loaded LCNP was in the range of 12.31% ± 0.41 to 14.12% ± 0.32 w/w. Compared with OAF2 (84.56% ± 0.19) and OAF3 (81.22% ± 0.15), OA loading LCNP (OAF1) showed the largest in vitro drug release rate of 85.49% ± 0.21 within 12 h as shown in Fig. 7. The in vitro release of OA from the LCNP dispersion was biphasic with an initial burst effect followed by a gradual release of OA. The initial release may be due to the presence of un-embedded drug in the LCNP dispersion, or it may be due to the presence of dissolved forms of the drug in the outer shell of the liquid lipid and lead to burst in the initial phase. However, release may occur due to corrosion or diffusion of the substrate [28]. The drug provided by the initial burst release immediately produces a rapid therapeutic effect, and the permeability of the drug was improved, while the sustained release of the drug is prolonged and the concentration of the therapeutic agent at the site of action is maintained. This clearly shows that sustained release can be obtained by using this formulation with a single application.

### Characterization of OA-LCNP-loaded topical gel

**Fig. 8:**
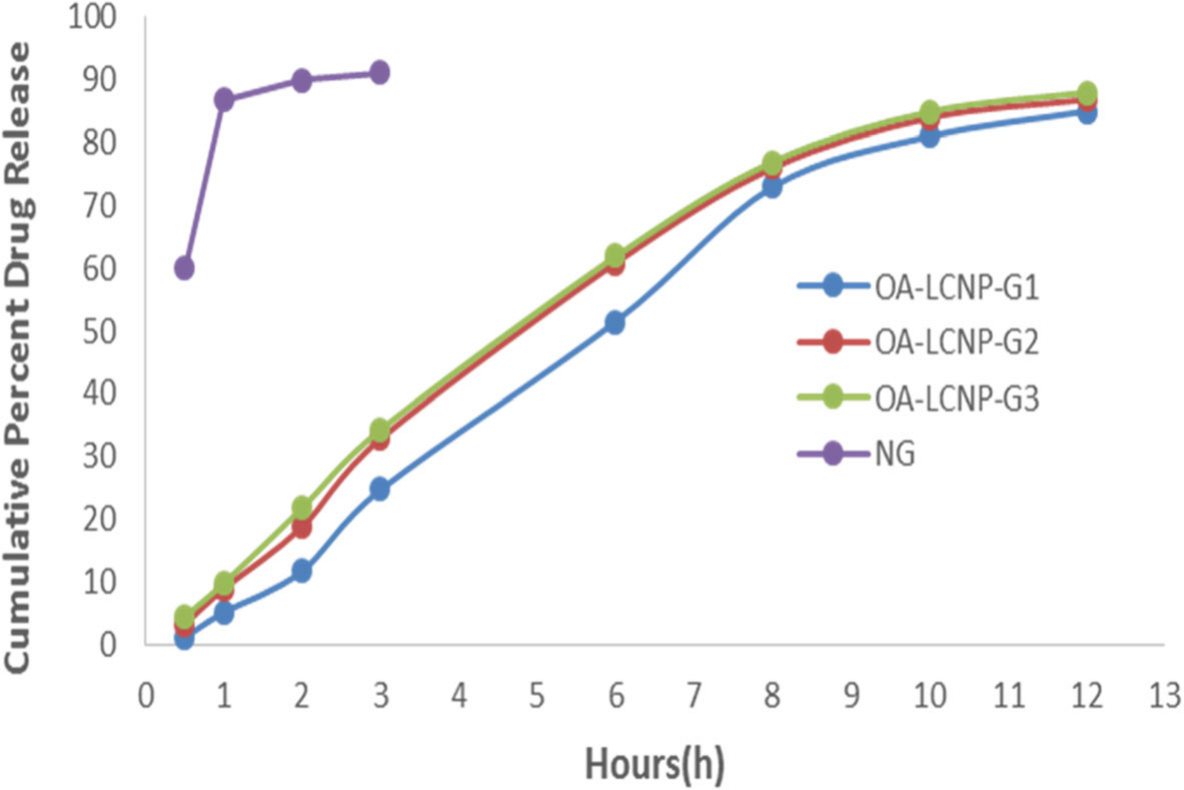
In vitro drug release profiles of OA-LCNP-loaded topical gels up to 12 h

The preparations of OA-LCNP-loaded topical gel were lucid, clear, and uniformed in texture, and the pH ranges of 7.1–7.4 which could easily be tolerated on skin without any stimulation. On account of lowest concentration of HPMC compared with OA-LCNP-G2 (64,000–71,000 cps) and OA-LCNP-G3 (55,000–63,000 cps), OA-LCNP-G1 had the lowest viscosity (72,000–79,000 cps). OA-LCNP-G1 can be spread easily for application compared to OA-LCNP-G2 since its slightly lower viscosity. Results for LCNP-loaded gel characterization are provided in Table 6. Values for percentage in vitro drug release up to 12 h for OA-LCNP-G1, G2, and G3 are 84.93, 86.78, and 87.89%, respectively, as given in Fig. 8.

**Table 6:** Characterization parameters for OA-LCNP-loaded gel

S.No.	Formulation	pH	Viscosity(cps)	Spreadability (g·cm·min^−1^)	Drug content (% w/w)
1.	OA-LCNP-G1	7.3	72,000 ~ 79,000	258.98/2.35	96.12 ± 0.31
2.	OA-LCNP-G2	7.4	64,000 ~ 71,000	241.33/2.43	97.32 ± 0.29
3.	OA-LCNP-G3	7.3	55,000 ~ 63,000	247.91/2.41	96.89 ± 0.12
4.	NG	7.4	67,000 ~ 73,000	246.38/3.13	97.46 ± 0.41

It was found that the in vitro release of the gel prepared by OA-LCNP was divided into two stages, the initial burst effect followed by the gradual release of OA. The initial burst may be due to the presence of unembedded drug in the LCNP system, or due to the liquid lipid located in the outer shell which contained the lipophilic drug in dissolved form and caused the initial burst. Release may occur through erosion or diffusion of the gel matrix [36]. The initial burst release provided immediate treatment and improved the permeability of the drug. The sustained release could ensure the stable concentration of the drug and maintain for a longer period of time.

**Fig. 9:**
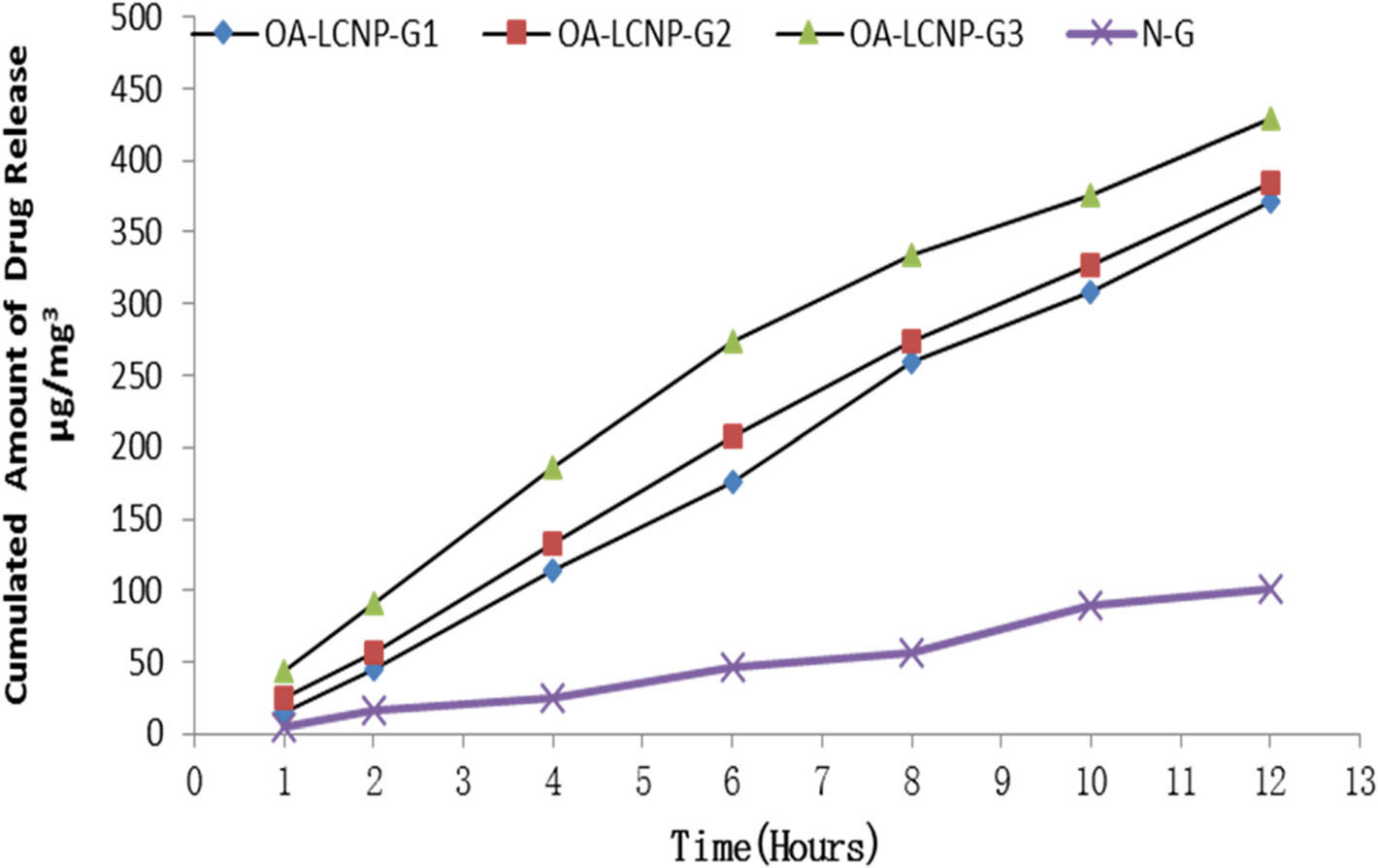
Ex vivo permeation results of different OA-LCNP-loaded gel formulations

The permeation study suggested that permeability parameters such as steady-state flux (Jss), permeability coefficient (Kp), and increased ratio were significantly higher in both of the OA-LCNP-G1 and OA-LCNP-G2 formulations, compared to OA-LCNP-G3. The cumulative amount of the permeated drug at the end of 12 h was 371.5, 384.7, and 429.8 μg/cm^2^ with a steady state flux (Jss) of 188.5, 195.2, and 218.0 μg/cm^2^/h for OA-LCNP-G1, OA-LCNP-G2, and OA-LCNP-G3, respectively, as shown in Fig. 9. Value of permeability coefficient is high for OA-LCNP-G1 (1.16 cm/h) > OA-LCNP-G3 0.96 cm/h) > OA-LCNP-G2 (0.98 cm/h). There was an increased ratio of OA-LCNP-G3 and OA-LCNP-G2, 1.16 and 1.36, respectively, compared to the OA-LCNP-G1 gel preparation. There were two processes in the infiltration study, including the initial burst effect followed by the gradual release of OA, which is consistent with previous in vitro release studies.

The in vitro drug release from the optimized OA-LCNP-G1 was best explained by Higuchi’s equation, as the plots showed the highest linearity (R^2^ = 0.989), followed by first order (R^2^ = 0.924) and zero order (R^2^ = 0.887). The corresponding plot of (log % cumulative drug release vs log time) for the Korsmeyer–Peppas equation indicated good linearity (R^2^ = 0.963). The value r^2^ of Peppas equation indicated good linearity showing irregular (non-Fickian) diffusion which means that drug release was controlled by more than one process, and the diffusion may be controlled as well as swelling controlled release as shown in Table 7.

**Table 7:** Release kinetic parameters for OA-loaded LCNP gel (OA-LCNP-G2)

Zero order		First order equation		Peppas equation		Higuchi equation	
*k*	*R*^*2*^	*k*	*R*^*2*^	*k*	*R*^*2*^	*k*	*R*^*2*^
3.124	0.887	0.0416	0.924	0.6048	0.963	18.212	0.989

### In vivo anti-inflammatory studies

The anti-inflammatory activity of the optimized preparation for OA-LCNP-loaded topical gel was evaluated using the carrageenan-induced hind paw inflammation method on Wistar rats. The percentage inhibition value of OA-LCNP gel (test) was also compared to marketed mometasone furoate gel (standard) as displayed in Table 8. OA-LCNP gel decreased the inflammation to a certain degree and also revealed a sustained effect for a prolonged period.

**Table 8:** In vivo anti-inflammatory activity: carrageenan-induced hind paw edema

Treatment	Anti-inflammatory activity	Time						
		0	1	2	4	8	12	24
Control	Edema rate	0.00 ± 0.02	34.12 ± 0.02	43.87 ± 0.02	44.32 ± 0.01	45.87 ± 0.02	46.98 ± 0.02	40.92 ± 0.02
Test	Edema rate	0.00 ± 0.01	32.28 ± 0.02	39.56 ± 0.02	36.81 ± 0.02	36.13 ± 0.02	35.41 ± 0.02	25.28 ± 0.02
	Inhibition rate(%)		5.61	7.81	17.32	20.83	24.89	45.26
Standard	Edema rate	0.00 ± 0.01	31.72 ± 0.02	34.11 ± 0.02	31.87 ± 0.02	26.13 ± 0.02	21.09 ± 0.02	17.42 ± 0.02

## Conclusions

In this study, OA-loaded LCNP was prepared by the precursor method, resulting in a well-characterized OA-LCNP gel preparation. The gel was shown to be effective in a rodent carrageenan-induced hind paw inflammation with sustained efficacy after a single application, but there is still a pressing need for further in vivo studies to realize their full potential.

## Data Availability

All data generated or analysed during this study are included in this published article.
